# Renal denervation does not affect hypertension or the renin-angiotensin system in a rodent model of juvenile-onset polycystic kidney disease: clinical implications

**DOI:** 10.1038/s41598-021-93575-0

**Published:** 2021-07-12

**Authors:** Sheran Li, Cara M. Hildreth, Ahmed A. Rahman, Sean A. Barton, Benjamin F. Wyse, Chai K. Lim, Paul M. Pilowsky, Jacqueline K. Phillips

**Affiliations:** 1 Department of Biomedical Sciences, Faculty of Medicine, Human and Health Sciences, Macquarie University, Sydney, NSW 2109 Australia; 2 Discipline of Physiology, School of Medical Sciences, University of Sydney, Sydney, Australia

**Keywords:** Polycystic kidney disease, Experimental models of disease, Cardiovascular diseases, Hypertension, Autonomic nervous system

## Abstract

We examined the effect of total and afferent renal denervation (RDN) on hypertension and the renin-angiotensin system (RAS) in a rodent model of juvenile-onset polycystic kidney disease (PKD). Lewis Polycystic Kidney (LPK) and control rats received total, afferent or sham RDN by periaxonal application of phenol, capsaicin or normal saline, respectively, and were monitored for 4-weeks. Afferent RDN did not affect systolic blood pressure (SBP) determined by radiotelemetry in either strain (n = 19) while total RDN significantly reduced SBP in Lewis rats 4-weeks post-denervation (total vs. sham, 122 ± 1 vs. 130 ± 2 mmHg, *P* = 0.002, n = 25). Plasma and kidney renin content determined by radioimmunoassay were significantly lower in LPK vs. Lewis (plasma: 278.2 ± 6.7 vs. 376.5 ± 11.9 ng Ang I/ml/h; kidney: 260.1 ± 6.3 vs. 753.2 ± 37.9 ng Ang I/mg/h, *P* < 0.001, n = 26). These parameters were not affected by RDN. Intrarenal mRNA expression levels of renin, angiotensinogen, angiotensin-converting enzyme (ACE)2, and angiotensin II receptor type 1a were significantly lower, whereas ACE1 expression was significantly higher in the LPK vs. Lewis (all *P* < 0.05, n = 26). This pattern of intrarenal RAS expression was not changed by RDN. In conclusion, RDN does not affect hypertension or the RAS in the LPK model and indicates RDN might not be a suitable antihypertensive strategy for individuals with juvenile-onset PKD.

## Introduction

Polycystic kidney disease (PKD) is a leading cause of end-stage renal disease, accounting for approximately 8% of patients receiving dialysis or renal transplantation in the United States^1^. Before progressing to end-stage renal disease, PKD patients commonly present with cardiovascular complications including hypertension, ischaemic heart disease and heart failure^2^. Excessive sympathetic nerve activity is believed to play an important role in the development of these cardiovascular complications^3^. Accumulating evidence also suggests the involvement of the renin-angiotensin system (RAS), both systemic and intrarenal, in the pathogenesis of PKD^4,5^. Intrarenal RAS refers to the local autocrine/paracrine system in the tissue, in this case the kidney, with all the components necessary to generate angiotensin II (Ang II) in situ^6^. Dysregulation of intrarenal RAS is described in human PKD, as evidenced by ectopic expression of renin in some cysts and dilated tubules^7–9^. Upregulation of intrarenal RAS components including renin, angiotensin-converting enzyme (ACE) and Ang II has been described in a rodent model of autosomal recessive PKD (ARPKD)^10^ (in the absence of any changes in systemic Ang) and similar findings were seen in mouse models with loss of cilia or polycystin 1, where cyst formation and increased blood pressure were associated with increased kidney angiotensinogen (AGT) levels^11^.

Complex interactions between the sympathetic nervous system and RAS activity exist in the kidney. Sympathetic nerves promote juxtaglomerular renin release in response to decreased blood pressure, driving systemic levels of Ang II^12^. There is also evidence they can impact intrarenal RAS, with renal denervation (RDN) reducing intrarenal renin protein and mRNA levels in a mouse model of neurogenic hypertension^13^. RDN also reduced blood pressure in these models.

In chronic kidney disease, data from preclinical^14^ and clinical studies^15^ has shown RDN can produce beneficial effects on blood pressure, cardiac and renal function. It is proposed that both sympathetic and sensory nerves are involved, as bilateral nephrectomy in chronic kidney disease patients reduces blood pressure and sympathetic activation, with the latter appearing to be mediated by an afferent signal arising in the diseased kidneys^16^. PKD would therefore seem to be an ideal candidate for RDN due to the presence of sympathetic activation and altered intrarenal RAS expression^3,10^. To date however, randomized clinical trials examining the impact of RDN in patients with PKD is lacking^17^, with only a limited number of case reports available, that do however, report a blood pressure lowering response^18,19^.

Both clinical and experimental RDN procedures typically destroy the renal sympathetic efferent and sensory afferent nerves; however, it is unclear whether the responses seen are due to the removal of sympathetic or afferent components, or both. The relative contribution may differ depending on the underlying disease aetiology. For e.g., the antihypertensive effect of RDN in the Dahl salt-sensitive model has been linked to the removal of sympathetic nerves^20^, whereas removal of afferent nerves is responsible for the response in the DOCA-salt hypertension model^21^.

The Lewis Polycystic Kidney (LPK) rat is a model of PKD arising from a mutation in Nek8, consistent with nephronophthisis (NPHP)9 in humans^22^. The model presents phenotypically similar to ARPKD, developing early-onset hypertension, alongside a progressive decline in renal and autonomic function^23,24^, and increased renal sympathetic nerve activity^25,26^. We have shown that systemic RAS is reduced in this model^23^, however, to date, the intrarenal RAS has not been studied.

In the present study we therefore tested the hypothesis that intrarenal RAS is upregulated in the LPK when compared to control rats, and that amelioration of this, through RDN, would limit the development of hypertension, cardiac autonomic and renal dysfunction. We undertook total RDN and selective sensory RDN as means to delineate the contribution of each component of the renal innervation to any outcomes observed.

## Results

### Effectiveness of RDN procedure

**Figure 1:**
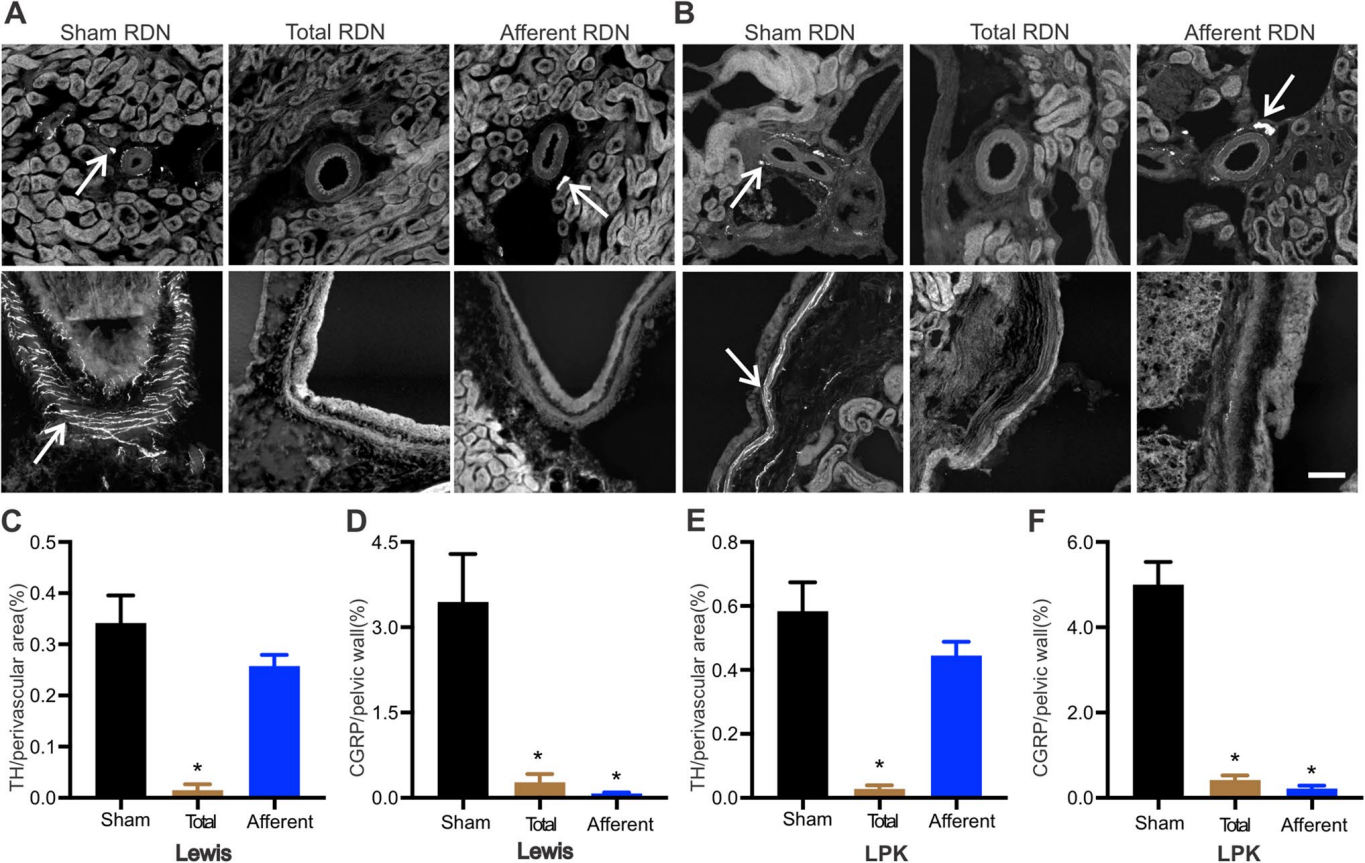
Validation of renal denervation (RDN) protocols determined using immunohistochemical assessment of tyrosine hydroxylase (TH) and calcitonin gene-related peptide (CGRP) innervation density one-week post-denervation as markers for sympathetic and sensory nerves respectively. Representative images of TH (top row) and CGRP (bottom row) staining in Lewis (**A**) and Lewis Polycystic Kidney (LPK; **B**) rats after sham, total and afferent RDN, respectively. Arrow indicates positive staining, scale bar lower right panel = 100 µm for all images. Panels (**C**–**F**) present quantitative analysis of TH and CGRP innervation density staining. Data is expressed as mean ± SEM, *indicates *P* < 0.05 difference vs. sham RDN analysed using one-way ANOVA and Bonferroni’s post hoc analysis. n = 4–6, per treatment group per strain.

In Study 1, we firstly confirmed using immunohistochemistry that both total and sensory RDN, performed in 6-week-old Lewis and LPK animals, could ablate the relevant nerve populations, examining the kidney tissue one week after surgery. Representative immunohistochemistry images of perivascular tyrosine hydroxylase (TH) and pelvic calcitonin gene related peptide (CGRP) labelling as markers of sympathetic and sensory innervation, respectively, from Lewis and LPK rats one week after the different denervation protocols (sham, total and afferent) and quantitative analysis are provided in Fig. 1A and B. Our labelling indicated that in both the Lewis and LPK animals, TH labelling was significantly reduced to ~ 4% of sham levels and CGRP labelling was reduced to ~ 8% of sham levels following total RDN, indicating the effectiveness of the stripping/phenol application protocol in destroying both the sympathetic and sensory innervation of the kidney. After afferent RDN, there was no significant change in the TH labelling in either the Lewis or LPK animals while CGRP labelling was reduced to less than 5% of sham levels, demonstrating selective destruction of afferent nerve fibres within the renal nerve plexus. Analysis of the kidney sections further confirmed the marked morphological changes seen in the LPK, with cystic lesions distributed throughout the renal parenchyma as we have previously described^23,27^.

We also assessed the impact of the RDN procedures on morphological and biochemical parameters in the above mentioned animals. One week after surgery, two-way ANOVA indicated that the kidney weight/body weight ratio was overall higher in the LPK compared with Lewis and that RDN had no effect (Lewis sham 0.9 ± 0.02 vs. total 1.0 ± 0.05 vs. afferent 0.9 ± 0.02%, LPK sham 7.2 ± 0.8 vs. total 5.1 ± 0.7 vs. afferent 5.3 ± 0.2%, ANOVA F = 130.5, *P* < 0.0001 between strains, F = 2.6, *P* = 0.10 between treatment groups, n = 28). Heart weight/body weight ratio was overall higher in LPK rats compared with Lewis controls but was unaffected by RDN procedures (Lewis sham 0.39 ± 0.02 vs. total 0.39 ± 0.04 vs. afferent 0.38 ± 0.01%, LPK sham 0.56 ± 0.04 vs. total 0.50 ± 0.03 vs. afferent 0.49 ± 0.06%; F = 18.2, *P* < 0.001 between strains, F = 0.7, *P* = 0.52 between treatment groups analysed using two-way ANOVA, n = 28). This pattern was also observed for plasma urea (Lewis sham 6.8 ± 0.5 vs. total 10.5 ± 3.0 vs afferent 7.0 ± 0.7 mmol/L; LPK sham 16.8 ± 4.0 vs. total 10.2 ± 1.2 vs. afferent 10.8 ± 0.7 mmol/L, F = 4.7, *P* = 0.04 between strains, F = 0.7, *P* = 0.51 between treatment groups, n = 27). No overall strain or treatment effect was observed for plasma creatinine (Lewis sham 19.8 ± 2.8 vs. total 19.7 ± 0.9 vs. afferent 24.5 ± 1.0 µmol/L; LPK sham 29.5 ± 13.0 vs. total 15.4 ± 1.9 vs. afferent 17.5 ± 2.4 µmol/L, F = 0.01, *P* = 0.92 between strains, F = 0.4, *P* = 0.64 between treatment groups, n = 27).

Urinalysis for urinary protein to creatinine (UPC) ratio was assessed in 3 Lewis animals and ≥ 3 LPK animals per treatment group. Urinary protein was only detected in 4 of 9 Lewis rats tested (range 0.09 to 0.32 g/L) and 5 of 12 LPK animals tested (range 0.07 to 0.58 g/L). Thus, insufficient numbers from each treatment cohort were available for a statistical comparison of creatinine normalised values between treatment groups.

### Effect of total or afferent RDN on cardiovascular and autonomic function parameters

In Study 2a, we determined the impact of RDN on cardiovascular and autonomic parameters for 4 weeks post surgery. Prior to statistical analysis of the impact of RDN procedure, the data set was analysed using a univariate general linear model to determine if there was an effect of sex and if this interacted with any treatment effect for the parameters of systolic blood pressure (SBP), diastolic blood pressure (DBP) and heart rate (HR). Overall, a sex effect in Lewis SBP and in LPK SBP and HR (all *P* < 0.05) was noted, with Lewis females having a higher SBP compared to male Lewis, and LPK females having a lower SBP and a higher HR compared with male LPK rats (Supplementary Fig. S2). However, no treatment × sex interaction was noted for the parameters of SBP, DBP and HR. Therefore, 24 h blood pressure values from animals of both sexes were pooled to test the effect of treatment within each strain.

**Figure 2:**
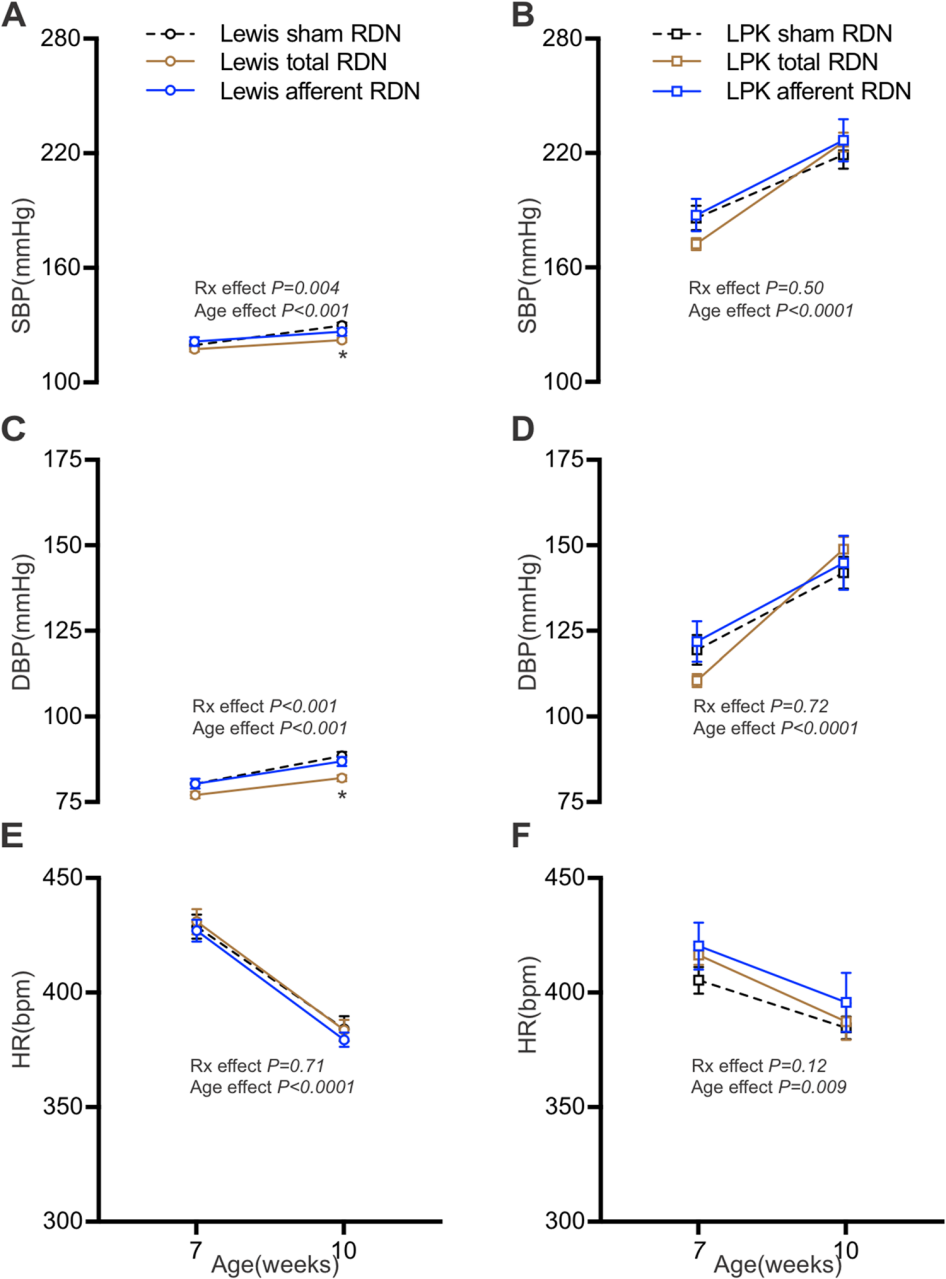
The effect of RDN on systolic blood pressure (SBP; **A**, **B**), diastolic blood pressure (DBP; **C**, **D**) and heart rate (HR; **E**, **F**) in Lewis (**A**, **C** and **E**) and LPK (**B**, **D** and **F**) rats at 7 and 10 weeks of age after sham, total and afferent RDN procedures. Rx, treatment effect. Data is expressed as mean ± SEM. *indicates *P* < 0.05 difference between sham and total RDN groups at 10 weeks old, analysed using two-way ANOVA and Bonferroni’s post hoc analysis. Overall age and treatment effects are provided in each panel. N values at 7 and 10 weeks for Lewis sham = (10, 11); Lewis total = (14, 13); Lewis afferent = (9, 10) respectively and for LPK sham = (13, 11), LPK total = (14, 12) and LPK afferent = (9, 8), respectively.

Consistent with the development of hypertension in the LPK reported previously^23^, SBP and DBP steadily increased in all LPK groups between the ages of 7 and 10 weeks (Fig. 2B,D), with no effect of either type of RDN on SBP and DBP observed relative to the sham controls (both *P* > 0.50). In the Lewis control group, SBP and DBP increased slightly with age (Fig. 2A,C). However, in contrast to our observations in the LPK, RDN lowered SBP (F = 6.1, *P* = 0.004), and DBP (F = 12.0, *P* < 0.001) in Lewis, with *post-hoc* analysis revealing that animals which had received total, but not afferent, RDN had a lower SBP and DBP at 10 weeks of age compared to sham animals (SBP, sham vs. total, 130 ± 2 vs. 122 ± 1 mmHg, *P* = 0.002; DBP, sham vs. total, 88 ± 1 vs. 82 ± 1 mmHg, *P* = 0.0002). In both strains, an age-related reduction in HR was observed (Fig. 2E,F). There was no treatment effect on HR in the Lewis or LPK animals (F = 0.3, *P* = 0.71 for Lewis, F = 2.5, *P* = 0.12 for LPK).

Diurnal variation in cardiovascular parameters for all Lewis and LPK animals is presented in Supplementary Fig. S3. In the Lewis, night-time (6 pm to 6 am) SBP and DBP was significantly higher than daytime (6 am to 6 pm) values (all *P* < 0.05), whereas in the LPK night-time and daytime SBP and DBP values were not significantly different (all *P* > 0.05). In both Lewis and LPK, night-time HR was significantly higher than daytime values (all *P* < 0.05). The effect of RDN on the diurnal variation of cardiovascular parameters, i.e., difference between night-time and daytime SBP (ΔSBP), DBP (ΔDBP) and HR (ΔHR) are presented in Supplementary Fig. S4. Neither total or afferent RDN affected these parameters within either strain (all *P* > 0.05).

**Figure 3:**
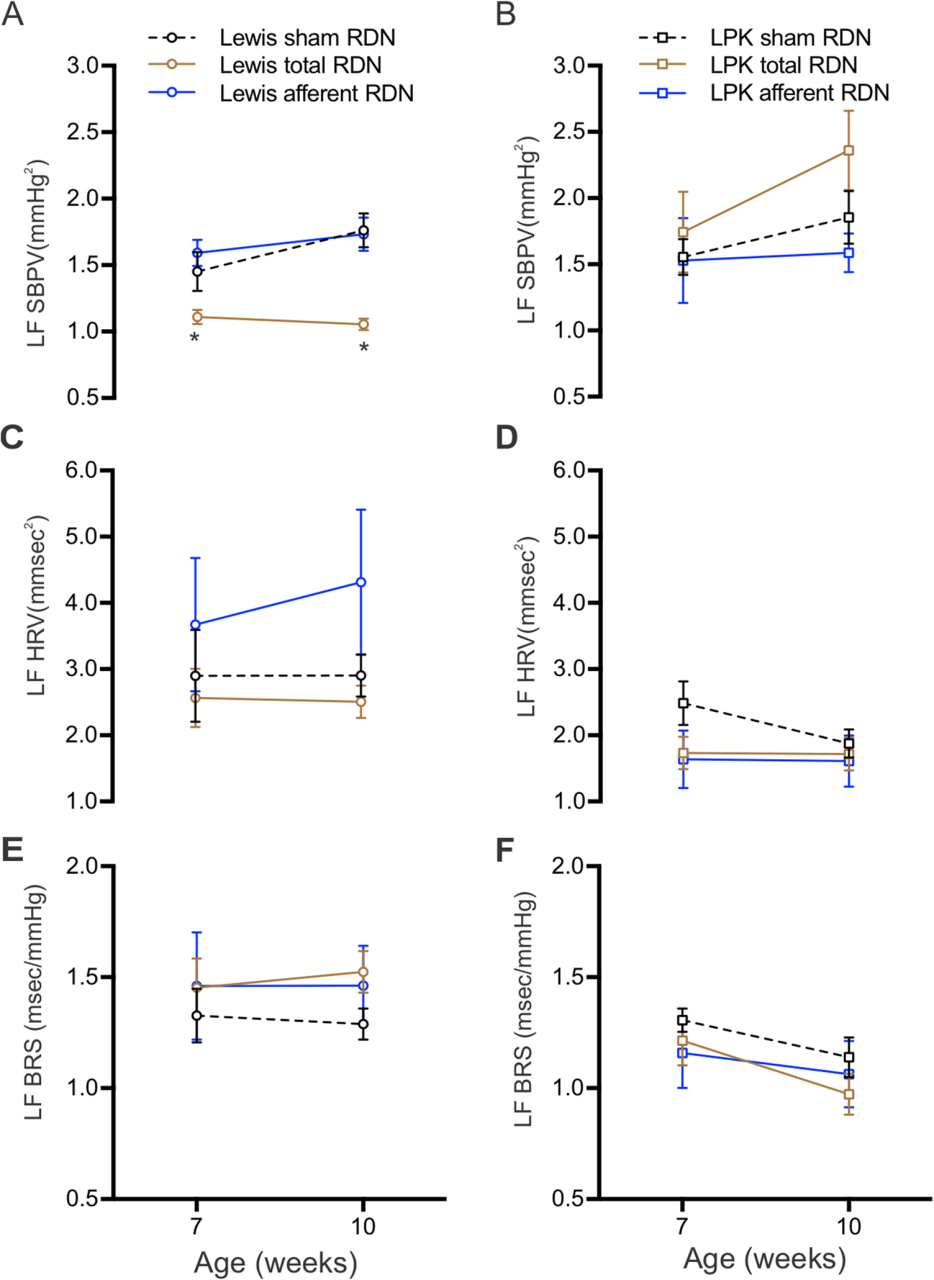
The effect of RDN on low frequency (LF) systolic blood pressure variability (SBPV; **A**, **B**), heart rate variability (HRV; **C**, **D**) and baroreceptor sensitivity (BRS; **E**, **F**) in Lewis (**A**, **C**, **E**) and LPK (**B**, **D**, **F**) rats at 7 and 10 weeks of age after sham, total and afferent RDN procedures. Data is expressed as mean ± SEM. *indicates *P* < 0.05 difference between sham and total RDN groups at 7 or 10 weeks old, analysed using two-way ANOVA and Bonferroni’s post hoc analysis. N values at 7 and 10 weeks for Lewis sham = (10, 11); Lewis total = (14, 13); Lewis afferent = (9, 10) respectively and for LPK sham = (13, 11), LPK total = (14, 12) and LPK afferent = (9, 8), respectively.

Cardiovascular autonomic function was assessed using power spectral analysis. In the Lewis, an overall treatment effect was noted in low frequency (LF) systolic blood pressure variability (SBPV) (Fig. 3A, F = 22.5, *P* < 0.0001), with total RDN producing a beneficial effect by lowering LF SBPV compared with sham RDN, evident at both 7 and 10 weeks old (*P* < 0.05). No treatment effect was noted for LF SBPV in LPK (Fig. 3B, F = 2.0, *P* = 0.15). There was also no effect on heart rate variability (HRV) (Fig. 3C,D) or baroreceptor sensitivity (BRS) (Fig. 3E,F) in Lewis and LPK (all *P* > 0.05).

### Effect of total or afferent RDN on systemic and intrarenal RAS

**Figure 4:**
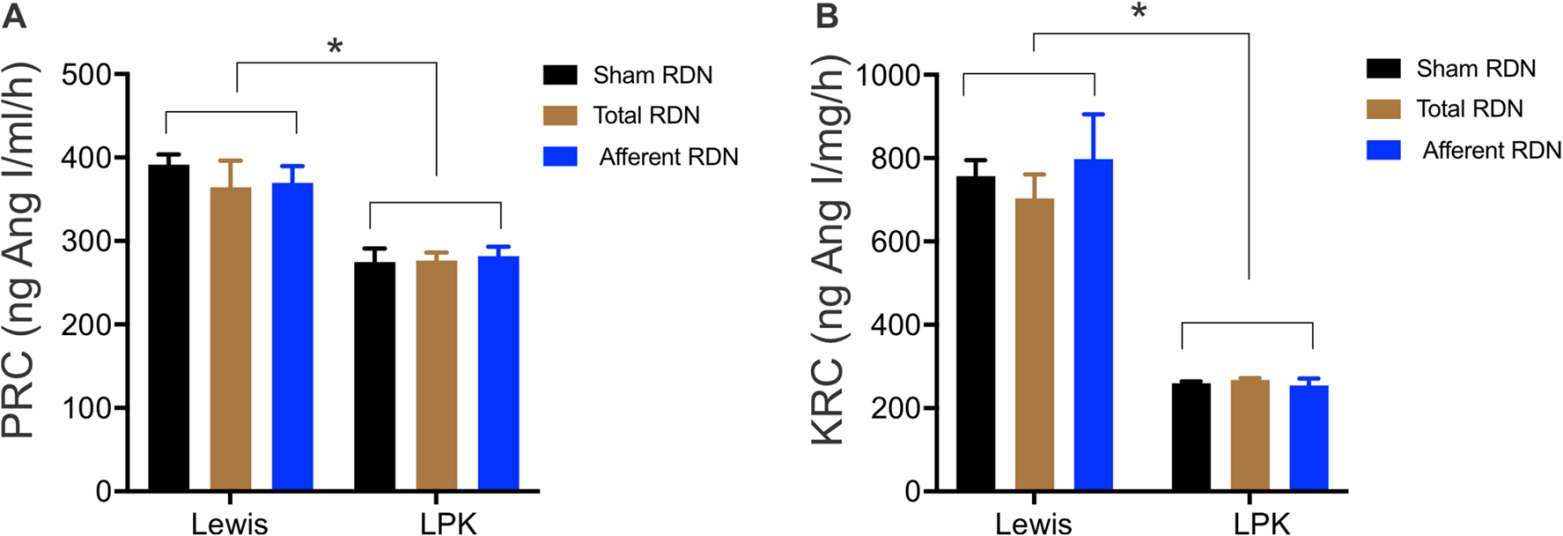
The effect of RDN on plasma renin content (PRC, **A**) and kidney renin content (KRC, **B**) in Lewis and LPK animals at 10 weeks of age. Data is expressed as mean ± SEM. *indicates *P* < 0.05 between strains as indicated, analysed using univariate general linear model analysis of variance. N = 4–5 per treatment per strain.

We then evaluated the impact of RDN on systemic and intrarenal RAS components in Lewis and LPK animals. Renin content in plasma and kidney samples was determined measuring the amount of Ang I generated per hour from added substrate. Plasma renin content was lower overall in the LPK compared with Lewis at 10 weeks of age (Lewis 376.5 ± 11.9 vs. LPK 278.2 ± 6.7 ng Ang I/ml/h; *P* < 0.0001) and RDN had no effect on this (*P* = 0.84; Fig. 4A). This pattern was also observed for kidney renin content (Lewis 753.2 ± 37.9 vs. LPK 260.2 ± 6.3 ng Ang I/mg/h; *P* < 0.0001) (Fig. 4B) where again RDN had no effect.

**Figure 5:**
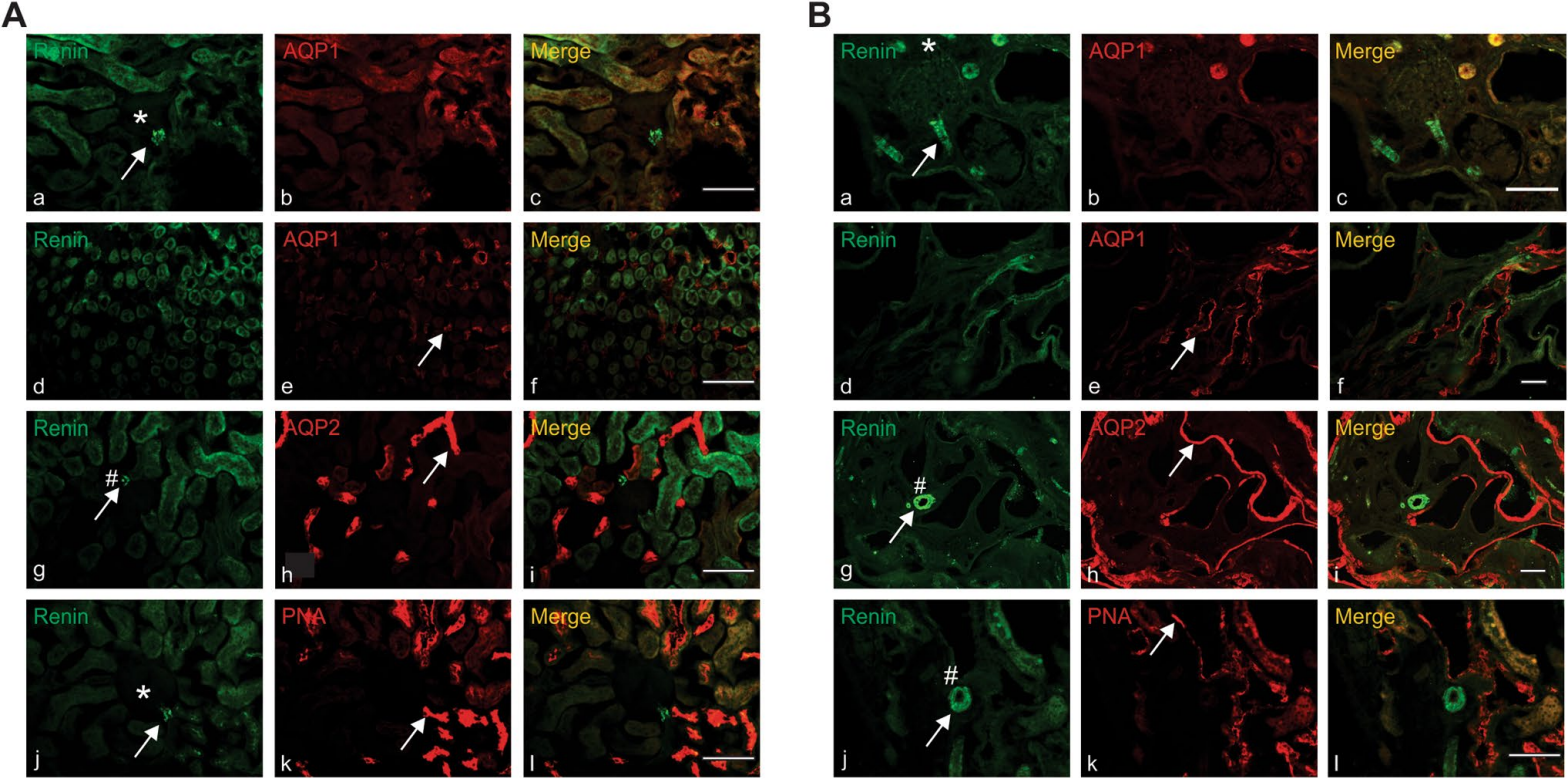
Representative images of double labelling of renin (a, d, g and j) and tubule segments marker AQP1 (b and e), AQP2 (h), PNA (k) in 7- or 10-weeks old Lewis (**A**) and LPK (**B**) rat kidney. Tubule segments marker AQP1 indicates the proximal tubule cells (b), AQP2 indicates the cortical and medullary collecting duct (e) and PNA indicates the distal tubule cells (h). Renin can be clearly seen in the juxtaglomerular apparatus (JGA, *indicates glomeruli) and blood vessels (#) clearly distinguishable from the low level autofluorescence in the renal tubules consistent with that previously described^58^. Arrows indicate positive staining and scale bar = 100 µm for all images.

Kidney renin expression was also assessed immunohistochemically in animals that underwent sham RDN procedure to determine if there were any differences in renin localisation between the two strains. A comparison of the two strains indicated a similar pattern of renin staining in the kidneys, which was evident in both the juxtaglomerular apparatus and vascular tissue, and was not colocalised with renal tubule markers (Fig. 5). Given the lack of effect of RDN on intrarenal renin content described above, the expression pattern of kidney renin was not assessed further.

Analysis of RAS gene expression in 10 week old Lewis and LPK animals indicated that angiotensinogen (AGT), renin, angiotensin-converting enzyme 2 (ACE2) and angiotensin type 1a receptor (Agtr1a) were lower and ACE1 expression greater in the LPK compared with Lewis (see Table 1). However, there was no overall treatment effect within each strain for all genes examined (Tables 2 and 3).

**Table 1:** Fold difference in kidney RAS gene expression between Lewis and Lewis Polycystic Kidney (LPK) rats at 10 weeks of age.

Gene	ΔΔCt (Lewis)	ΔΔCt (LPK)	Fold difference v. Lewis (range)	P value
Renin	0.0 ± 0.4	1.5 ± 0.2	0.34 (0.29–0.40)	0.014
AGT	0.0 ± 0.6	3.2 ± 0.3	0.11 (0.09–0.14)	< 0.001
ACE1	0.0 ± 0.3	-1.6 ± 0.2	3.08 (2.73–3.47)	< 0.001
ACE2	0.0 ± 0.2	4.0 ± 0.3	0.06 (0.05–0.08)	< 0.001
Agtr1a	0.0 ± 0.1	0.7 ± 0.2	0.61 (0.52–0.72)	0.013

*RAS* renin-angiotensin system, *AGT* angiotensinogen, *ACE* angiotensin-converting enzyme, *Agtr1a* angiotensin type 1a receptor. Data are presented as ΔΔCt with the Lewis animals used as the reference group. Fold difference (range) was calculated using the 2^−(ΔΔCt ± Standard Error (SE) of the ΔCt)^ method.

**Table 2:** Fold difference in kidney RAS gene expression in Lewis rats after RDN.

Gene	ΔΔCt			Fold difference v. sham (range)		P value
	Sham RDN	Total RDN	Afferent RDN	Total RDN	Afferent RDN	
Renin	0.0 ± 0.7	0.1 ± 0.7	0.8 ± 0.8	0.92 (0.56–1.51)	0.59 (0.33–1.05)	0.75
AGT	0.0 ± 1.1	− 0.6 ± 1.2	-0.9 ± 1.1	1.51 (0.65–3.50)	1.81 (0.87–3.76)	0.86
ACE1	0.0 ± 0.6	− 0.5 ± 0.6	0.1 ± 0.4	1.46 (0.9–2.24)	0.92 (0.72–1.19)	0.68
ACE2	0.0 ± 0.3	− 0.5 ± 0.3	-0.5 ± 0.4	1.38 (1.10–1.73)	1.45 (1.10–1.92)	0.50
Agtr1a	0.0 ± 0.2	0.1 ± 0.2	− 0.1 ± 0.2	0.93 (0.82–1.06)	1.05 (0.91–1.22)	0.84

*RAS* renin-angiotensin system, *AGT* angiotensinogen, *ACE* angiotensin-converting enzyme, *Agtr1a* angiotensin type 1a receptor. Data are presented as ΔΔCt with the sham animals used as the reference group. Fold difference (range) was calculated using the 2^−(ΔΔCt ± Standard Error (SE) of the ΔCt)^ method.

**Table 3:** Fold difference in kidney RAS gene expression in Lewis Polycystic Kidney (LPK) rats after RDN.

Gene	ΔΔCt			Fold difference vs. sham (range)		P value
	Sham RDN	Total RDN	Afferent RDN	Total RDN	Afferent RDN	
Renin	0.0 ± 0.2	− 0.3 ± 0.2	− 0.3 ± 0.5	1.27 (1.10–1.46)	1.19 (0.81–1.75)	0.84
AGT	0.0 ± 0.6	0.6 ± 0.4	− 1.0 ± 0.5	0.67 (0.51–0.87)	2.07 (1.49–2.87)	0.10
ACE1	0.0 ± 0.1	0.2 ± 0.2	0.4 ± 0.4	0.87 (0.75–1.02)	0.74 (0.56–0.99)	0.64
ACE2	0.0 ± 0.4	− 0.1 ± 0.2	− 1.0 ± 0.5	1.07 (0.95–1.20)	2.00 (1.43–2.82)	0.20
Agtr1a	0.0 ± 0.3	− 1.0 ± 0.4	− 0.3 ± 0.5	1.98 (1.47–2.65)	1.22 (0.89–1.68)	0.25

*RAS* renin-angiotensin system, *AGT* angiotensinogen, *ACE* angiotensin-converting enzyme, *Agtr1a* angiotensin type 1a receptor. Data are presented as ΔΔCt with the sham animals used as the reference group. Fold difference (range) was calculated using the 2^−(ΔΔCt ± Standard Error (SE) of the ΔCt)^ method.

### Effect of total or afferent RDN procedure on morphometric and biochemical parameters

The morphometric and biochemical parameters for animals at age 10 weeks are shown in Table 4. For this cohort of animals, two-way ANOVA indicated that the LPK had an overall higher level of kidney weight /body weight ratio, heart weight, heart weight/ body weight ratio, 24 h water intake, urine output and plasma urea compared with Lewis (all *P* < 0.01) as well a lower creatinine clearance rate [(CCR) Lewis 2.0 ± 0.3 vs. LPK 1.0 ± 0.2 ml/min, *P* = 0.004, N = 24], confirming impairment of renal function and cardiac hypertrophy at this age. Urine protein was below the level of detection in 4 of 13 Lewis rats tested meaning insufficient numbers from each treatment cohort were available for a statistical comparison of UPC between treatment groups however UPC was higher overall in the LPK (both *P* < 0.001), confirming filtration barrier injury in the LPK. Urinary sodium measured over 24 h was not significantly different between LPK and Lewis (F = 2.2, *P* = 0.16). There was no treatment effect of RDN in any of these parameters in either strain (all *P* > 0.05, exact P values presented in Table 4).

**Table 4:** Morphometric and biochemical parameters at 10 weeks of age.

	Sham RDN	Total RDN	Afferent RDN	Treatment effect
**Lewis (n)**	(5)	(4)	(4)	
KW/BW ratio (%)	0.8 ± 0.02	0.8 ± 0.04	0.8 ± 0.01	0.88, 0.43
Heart weights (g)	0.79 ± 0.07	0.82 ± 0.08	0.92 ± 0.11	1.6, 0.23
HW/BW ratio (%)	0.33 ± 0.01	0.33 ± 0.01	0.35 ± 0.02	2.3, 0.12
24 h water intake (ml)	18.2 ± 4.1	21.2 ± 2.6	23.8 ± 2.3	0.03, 0.97
24 h urine output (ml)	8.5 ± 1.7	10.2 ± 0.9	10.4 ± 1.8	0.14, 0.87
UPC	0.2 ± 0.0	(0.19/0.13)#	(0.07/0.39)^#^	n/a, n/a
CCR (ml/min)	2.8 ± 0.6	2.1 ± 0.3	2.3 ± 0.5	0.22, 0.80
Plasma urea (mmol/L)	7.0 ± 0.5	8.3 ± 1.0	7.3 ± 0.4	0.72, 0.50
Plasma creatinine (µmol/L)	24.2 ± 1.9	38.5 ± 10.4	27.5 ± 2.5	0.29, 0.75
24 h urine sodium (mmol)	1.6 ± 0.5	2.3 ± 0.2	1.6 ± 0.2	2.11, 0.15
**LPK (n)**	(3–4)	(4)	(3–5)	
KW/BW ratio (%)	8.3 ± 0.5	7.4 ± 0.7	8.8 ± 0.8	0.88, 0.43
Heart weights (g)	0.98 ± 0.06	0.90 ± 0.07	1.05 ± 0.05	1.6, 0.23
HW/BW ratio (%)	0.54 ± 0.03	0.46 ± 0.05	0.57 ± 0.04	2.3, 0.12
24 h water intake (ml)	40.5 ± 1.0	39.1 ± 3.4	35.8 ± 4.6	0.03, 0.97
24 h urine output (ml)	27.4 ± 1.7	26.6 ± 2.8	23.6 ± 5.2	0.14, 0.87
UPC	5.2 ± 1.5	2.7 ± 0.4	8.6 ± 6.7	n/a, n/a
CCR (ml/min)	1.2 ± 0.4	1.6 ± 0.3	1.0 ± 0.3	0.22, 0.80
Plasma urea (mmol/L)	26.5 ± 8.6	15.6 ± 0.7	24.1 ± 6.5	0.72, 0.50
Plasma creatinine (µmol/L)	65.7 ± 33.2	32.5 ± 4.1	71.0 ± 30.2	0.29, 0.75
24 h urine sodium (mmol)	1.4 ± 0.2	1.7 ± 0.1	1.3 ± 0.1	2.11, 0.15

RDN, renal denervation. KW/BW, kidney weight/body weight. HW/BW, heart weight/body weight. UPC, urine protein to creatinine ratio. CCR, creatinine clearance rate. Data is expressed as the mean ± SEM. ^#^In the Lewis, 2 animals in the total RDN and 2 animals in the afferent RDN group had urine protein levels below the level of detection. Thus the values for UPC are provided as each animals individual result and no statistical analysis was undertaken. Treatment effect is provided as F statistic and *P* value, indicating overall treatment effect determined using two-way ANOVA with strain and treatment as the two variables.

### Innervation profile

**Figure 6:**
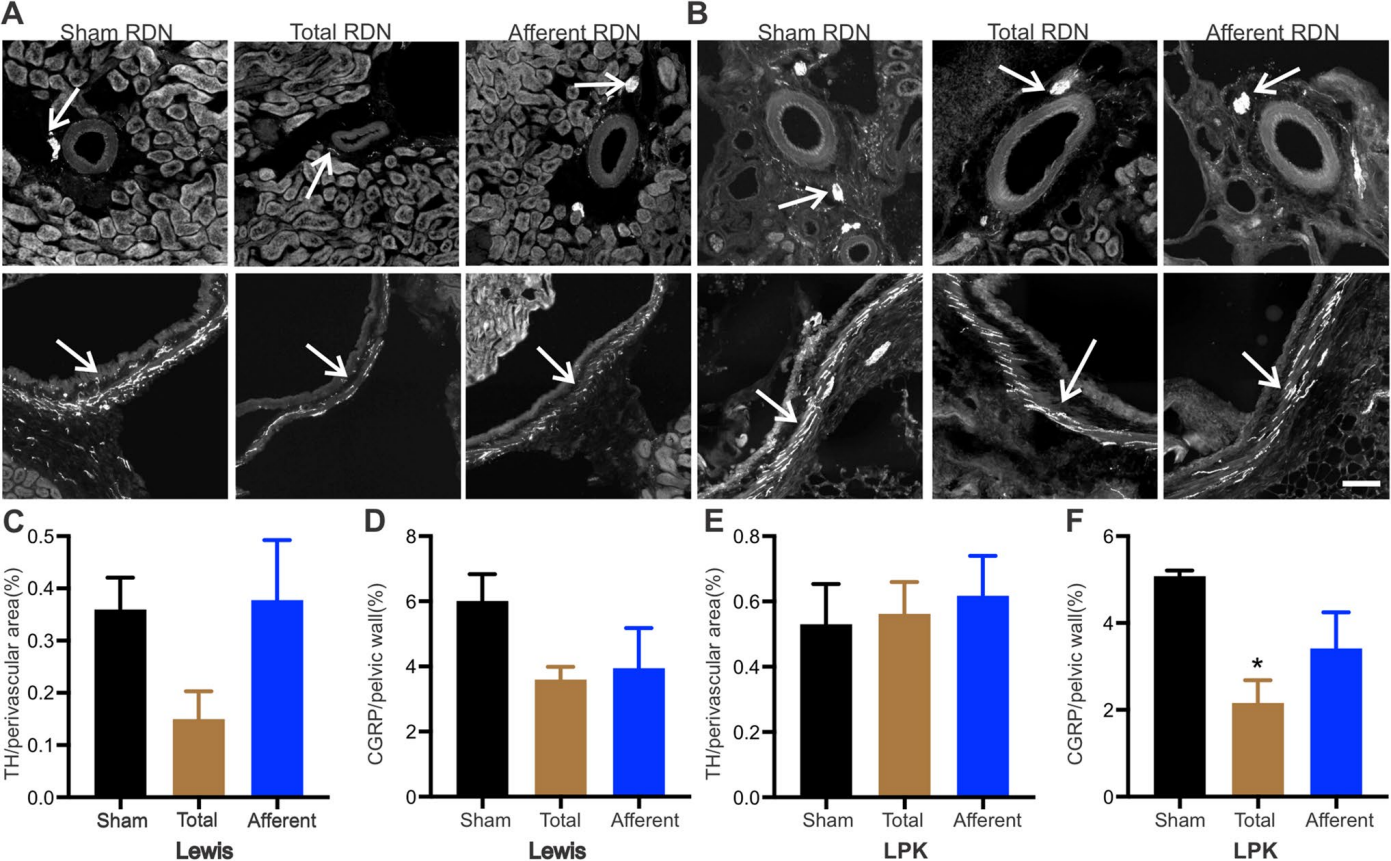
Renal reinnervation by immunohistochemical assessment of tyrosine hydroxylase (TH) and calcitonin gene-related peptide (CGRP) innervation density four-week post-denervation as markers for sympathetic and sensory nerves, respectively. Representative images of TH (top row) and CGRP (bottom row) staining at 4 weeks post sham, total and afferent RDN in the Lewis (**A**) and LPK (**B**). Arrows indicate positive staining and scale bar in lower panel = 100 µm for all images. Quantitative analysis is shown in panel (**C**–**F**). Data is expressed as mean ± SEM. *indicates *P* < 0.05 difference vs. sham RDN, analysed using one-way ANOVA and Bonferroni’s post hoc analysis. N = 4–5, per treatment group per strain.

Based on studies that show sympathetic and afferent renal reinnervation occurs in the rat after renal denervation^28,29^ we examined the kidneys of the animals immunohistochemically as detailed for Study 1 to determine to what degree this had occurred. Representative images of perivascular TH and pelvic CGRP labelling from Lewis and LPK at age 10 weeks (4 weeks after the different denervation protocols) are shown in Fig. 6. After total RDN in the Lewis, at 10 weeks, both TH and CGRP nerve density were not statistically different compared to sham animals (F = 2.5, *P* = 0.13 and F = 2.3, *P* = 0.15 respectively), indicating reinnervation was well established. After afferent RDN in the Lewis at 10 weeks, there was no significant difference from sham animals for TH (F = 2.5, *P* = 0.13), as anticipated, but also for CGRP levels (~ 66% of sham levels, F = 2.3, *P* = 0.15), indicating significant sensory reinnervation had occurred after this procedure. In the LPK, after total RDN, there was no significant difference between sham and denervated animals for TH at age 10 weeks (~ 65% of sham levels, F = 0.2, *P* = 0.86). However, CGRP levels were significantly less (~ 42% of sham, F = 5.0, *P* = 0.03 with post hoc analysis showing *P* = 0.02). After afferent RDN, there was no significant difference from sham animals for TH (F = 0.2, *P* = 0.86), again as anticipated, but also for CGRP levels (~ 67% of sham, *P* = 0.19 by post hoc analysis), indicating significant sensory reinnervation having occurred as seen in the Lewis.

## Discussion

The major findings of our study are that neither total nor afferent RDN affects blood pressure, autonomic or renal function in LPK animals and that total RDN, but not selective afferent ablation, produces a modest blood pressure reduction in Lewis control animals. In addition, intrarenal renin content, as well as mRNA for intrarenal RAS components, were significantly less in LPK vs. Lewis control animals, and this was not altered within either strain by either total or afferent denervation procedures. Lastly, anatomical evidence of regrowth of the renal efferent and afferent nerves was present at 4 weeks post total-RDN as detected by immunohistochemistry.

Renal sympathetic nerves play a tonic role in the regulation of blood pressure under normotensive conditions^30^ and consistent with this, in the present study total RDN produced a modest reduction in systolic and diastolic blood pressure in the Lewis that was of comparable magnitude to that observed in other normotensive animal models after RDN^31^. We also observed an overall reduction in LF SBPV in the Lewis controls that received total RDN, evident within one week of the denervation, before the reduction of blood pressure. As the LF component of SBPV is contributed to by sympathetic modulation in rats^32^, a reduction in LF SBPV indicates a decrease in the sympathetic branch of autonomic cardiovascular control^32^, and suggests that a reduction in vasomotor tone underlies the blood pressure-lowering effect of total RDN.

We further explored the mechanism by which total RDN reduced blood pressure in the Lewis animals by investigating if there were any changes in urine sodium excretion, plasma renin content or intrarenal components of the RAS following total RDN, all being important regulators of blood pressure^6,30^. However, total RDN did not alter urinary sodium excretion, plasma or kidney renin content or gene expression levels of intrarenal RAS components in the Lewis strain. This is consistent with a previous study in which total RDN reduced blood pressure without affecting plasma renin activity in normotensive Sprague–Dawley rats^33^. It is possible that any initial response to RDN resulting in differences in sodium excretion relative to sham animals was corrected by pressure-natriuresis mechanisms by the time the 24 h urine samples were collected at age 10 weeks. It is also important to note that due to technical limitations, we measured 24 h urinary sodium excretion instead of the fractional excretion of sodium, which is the standard method for sodium excretion determination^34^. Further, we did not monitor 24 h sodium intake and output. Nevertheless, these findings do suggest that alterations in sodium excretion or intrarenal RAS are not a primary driver of the sustained decrease in blood pressure produced by total RDN in control rats.

A key finding of our study was that neither total or sensory RDN affected blood pressure or heart weight ratio in the LPK model of juvenile-onset PKD. This is in contrast to the only other published study examining renal denervation in a PKD animal model, where using Han: SPRD-Cy/ + rats, total RDN was shown to reduce blood pressure^35^. There are a number of possible explanations for our contrasting observations. Firstly, unlike the Han: SPRD-Cy/ + PKD model, which presents with mild hypertension, the LPK model displays marked hypertension^23,24^ alongside elevated renal sympathetic nerve activity at a young age^36^. Noting the early onset of hypertension in these animals, it is conceivable that the timing of the RDN intervention in our study was not early enough to limit the effect of increased renal sympathetic nerve activity on the development or progression of hypertension in the LPK. Secondly, a positive association between blood pressure and renal size has been described in children, adolescents and adults with PKD^37,38^. Kidney volume was reduced in the Han: SPRD-Cy/ + model after total RDN^35^, but likely remained unchanged after RDN in our model as evidenced by no difference in kidney weight to body weight ratios when compared to the sham procedure. Notably, in the human studies the relationship between blood pressure and kidney size was found to be independent of the degree of renal dysfunction. In our study, neither total nor afferent RDN improved renal function. Within the context of PKD, the ability of RDN to limit disease progression is therefore likely dependent on the specific disease phenotype, including age of onset and cystic disease severity.

The LPK model of PKD exhibits marked autonomic dysfunction including increased cardiac sympathetic and reduced cardiac vagal regulation, and a reduction in cardiac baroreflex control that contribute to an overall increase in SBPV and reduction in HRV^24^. Unlike in the spontaneously hypertensive rat, where RDN has been shown to significantly improve cardiac and sympathetic baroreflex sensitivity independent of a blood pressure response^39,40^, neither total nor afferent RDN affected HRV, SBPV or BRS in the LPK. This suggests that in this model, other pathways drive the perturbed autonomic dysfunction that is evident.

Activation of the RAS, both systemic and intrarenal, have been proposed as mechanisms for hypertension development in PKD^4,5^. However, in the current study, we demonstrated reduced systemic renin content, agreeing with our previous studies that demonstrated reduced plasma renin activity in this model^23^, and, in contrast to our hypothesis, we demonstrated lower levels of kidney renin content and intrarenal expression of the RAS component genes (with the exception of ACE1 mRNA) compared to our control animals. This is in contrast to the *pck* rat model of ARPKD^10^, where increased mRNA expression of renin and Ang II staining in the kidney was observed, and human autosomal dominant PKD^7,8^ and ARPKD^9^, where ectopic expression of renin in cysts was observed. The mechanism responsible for the suppressed systemic renin in our model may well be a consequence of the severe hypertension (~ 230 mmHg vs ~ 150 mmHg in the *pck* rat at a similar age), given that pressure dependent feedback on juxtaglomerular renin secretion through this classical pathway is linked to increased systemic and renal reperfusion pressures^41^. The mechanism responsible for reduced intrarenal RAS is not clear from the results of our study, noting further, and again contrary to our hypothesis, total RDN did not have any significant effect on any of the components we assessed. This may be because any effect of RDN on intrarenal RAS was counteracted by other pathways involved in intrarenal RAS regulation such as the prorenin receptor, Wnt/*β*-catenin signaling, prostaglandins, Klotho and nuclear receptors (as reviewed by^6^). This is an area warranting further study, noting that in this model, despite low RAS levels, both systemically and as we have now established, intrarenal, RAS inhibition is an effective blood pressure lowering strategy^25,41–44^.

An important consideration in the interpretation of this data is that the collection of blood samples was taken in animals after the administration of anaesthetic. Anaesthesia is known to activate the intrarenal RAS^45^ and as such, changes in our indicators of intrarenal RAS in response to RDN may have been masked. In the literature, the impact of RDN on both systemic and intrarenal RAS has shown to be variable. Following RDN, a decrease in plasma renin activity has been shown in a rabbit model of hypertension secondary to chronic kidney disease^14^ and a decrease in renal renin is seen in a mouse model of neurogenic hypertension^13^, while in the spontaneously hypertensive rat model of essential hypertension, total RDN had no effect plasma renin activity or kidney renin content^46^. In human clinical studies, RDN similarily had no impact on circulating renin in patients with resistant hypertension^47^. This suggests that the variable effects of RDN on RAS, both systemic and intrarenal, are mediated by the underlying mechanism in the different disease states.

A final aspect of our study was evidence of reinnervation in both Lewis and LPK rats after the RDN procedures. This is consistent with reports of both functional and anatomical reinnervation of the rat kidney by 4 weeks post-RDN^28,29^. While direct evidence of nerve regrowth after RDN in humans is lacking, renal sympathetic nerve regrowth is observed at 5 months post kidney transplantation^48^ and in a sheep model of catheter-based renal denervation, 11 months post procedure there is both functional and anatomical evidence of reinnervation^49^. What is unclear is whether renal reinnervation following RDN has any impact on the observed blood pressure response. In the present study, despite anatomical evidence of renal sympathetic and sensory nerves 4 weeks post procedure, the blood pressure of Lewis rats that underwent total RDN was still significantly lower than sham animals. If renal reinnervation does not dampen the initial blood pressure lowering effect, it would explain why a sustained response to catheter-based RDN lasting 3 years after the initial procedure has been observed in clinical studies^50^. This is also consistent with the spontaneously hypertensive rat model, where a second RDN procedure does not further reduce blood pressure^51^. An important caveat when interpreting our results, however, is while results using immunohistochemistry are qualitatively quite clear, due to the cystic features of the LPK kidneys, we were unable to apply unbiased stereology methods using random sections from the whole kidney. Our must therefore be considered as a semi-quantitative measure and cannot be directly translated into a complete loss of either innervation or functionality. Additional measures such as tissue levels of CGRP or noradrenaline, and functional validation of the denervation, for example, as undertaken in the study by Booth et al. in a sheep model of RDN^49^ would add to the strength of evidence. Another limitation of these studies is the sample numbers for both these groups (Studies 1 and 2b), which are relatively small. A posthoc power analysis indicated that the study power is over 80% for each one however which would indicate additional animals would be unlikely to alter the result.

The clinical implications of our work should be considered in context of the conflicting results surrounding clinical trials using catheter-based RDN studies^50,52^. It is crucial to determine which patient populations will benefit from the approach and, just as importantly, those which will not respond, thereby preventing unnecessary invasive procedures. Our study has demonstrated that neither total nor afferent RDN has a positive effect on blood pressure, autonomic function or renal function in a juvenile onset rodent model of PKD, suggesting that this procedure would not benefit patients suffering from comparable juvenile onset ARPKD or NPHP. Alternative therapeutic approaches to limit hypertension and renal dysfunction will be required.

## Materials and methods

All experiments were approved by the Animal Ethics Committee of Macquarie University and conducted in accordance with the Australian code for the care and use of animals for scientific purposes (8th Edition). The study was conducted and reported according to the ARRIVE guidelines. Lewis and LPK rats of both sexes (n = 33 male Lewis, n = 22 female Lewis, n = 32 male LPK, n = 29 female LPK, total = 116) were obtained from the Animal Resource Centre, Perth, Australia and housed at the Central Animal Facility of Macquarie University on a 12-h light–dark cycle (lights on 6 am), at 22 ± 2 °C with access to food and water  *ad libitum*. At the end of the study, animals were euthanised with an i.p. injection of 20% (v/v) solution of sodium pentobarbital (100 mg/kg, Virbac, NSW, Australia) prior to the collection of blood and tissues.

### Experimental protocols and study groups

The experimental protocol and study groups are illustrated in Supplementary Fig. S1. At age 6 weeks, all animals were subjected to one of three surgical procedures: (i) total RDN by periaxonal application of 10% phenol in ethanol^29^; (ii) afferent RDN by periaxonal application of 33 mM capsaicin^29^; or (iii) sham RDN by periaxonal application of normal saline. In Study 1, animals were euthanised one-week post-denervation or sham procedure (age 7 weeks), and kidneys collected for immunohistochemical assessment of renal sensory and sympathetic nerve innervation (total n = 13 Lewis and n = 15 LPK). In Study 2a, animals were instrumented with radiotelemetry probes and blood pressure and HR monitored to 10 weeks of age (total n = 35 Lewis and n = 36 LPK). In Study 2b, some of the animals (n = 9) from Study 2a and an additional group of animals were euthanised at age 10 weeks and biological samples including urine, plasma, heart and kidneys collected for subsequent analyses (total n = 13 Lewis and n = 13 LPK), including immunohistochemistry to assess what degree renal sensory and sympathetic nerve re-innervation had occurred at the end of the study period. A full breakdown of total animal numbers and sex used in each Study is provided in Supplementary Table S1, noting that specific numbers for each data set are provided as not all samples or recordings were available for every parameter from every animal. For example, in study 2a, signal loss associated with the probe, battery life or catheter patency precluded data collection across the full study protocol or in Study 2b, insufficient plasma was available to undertake urea, creatinine and renin determinations.

#### Anaesthesia

For all surgical procedures, anaesthesia was induced with 5% isoflurane in 100% O_2_ and animals were maintained on 2–3% isoflurane in 100% O_2_. Pre-operative analgaesia (carprofen; 2.5 mg/kg s.c. Norbrook Laboratories, VIC, Australia) and antibiotics (cephazolin; 50 mg/kg i.m. Hospira, VIC, Australia) were administered.

#### Renal denervation

Bilateral midline flank incisions were made to expose the right and left renal artery and vein. For total RDN*,* the renal arteries were stripped of all visible nerve bundles and painted with 10% phenol in absolute ethanol; for selective afferent RDN the renal artery was painted with 33 mM capsaicin (Sigma-Aldrich, MO, USA) dissolved in 5% ethanol and 5% Tween 80 in normal saline 2–3 times at 2–3 min intervals. Sham surgery entailed visualisation of the renal artery and painting with normal saline. Animals were provided with postoperative analgaesia (buprenorphine; 50 μg/kg, s.c Reckitt Benckiser Ltd, Auckland, New Zealand, immediately after the surgery, and then carprofen; 2.5 mg/kg s.c.) and supplementary fluids were administered once daily for 24–48 h postoperatively if required.

#### Telemetry probe implantation

At the time of the RDN procedure, animals in Study 2a had radiotelemetry probes (PA-C10 or HD-X10, Data Sciences International, MN, USA) implanted into the aorta via the right femoral artery with subcutaneous device placement. Animals were allowed to recover for 7–10 days before any radiotelemetry recordings were made.

#### Cardiovascular autonomic function analysis

Arterial pressure (AP) was continuously recorded for 5 min every 15 min for 48 h once weekly from 7 to 10 weeks of age (i.e., 192 recordings in total per week for 4 weeks). From the AP waveform, SBP, DBP and HR were derived offline using Dataquest ART (Data Sciences International) and exported as text into Microsoft Excel (Microsoft, WA, USA). All recordings over the 48 h period were averaged to create a mean 24 h value for analysis. For assessment of diurnal variation, all recordings during the daytime (6 am to 6 pm) or night-time (6 pm to 6 am) were averaged to create a mean daytime or night-time value of cardiovascular parameters.

To assess HRV, SBPV and BRS, which are surrogate markers of cardiovascular autonomic function, all AP waveforms acquired during the 48 h recording were imported into Spike 2 (version 7.02; Cambridge Electronic Designs, Cambridge, UK) and the segments between 11 am and 1 pm (day) and 11 pm and 1 am (night) were chosen for subsequent analysis. The pulse interval was derived from the AP waveform and uniformly resampled at 10 Hz. 80 s segments of AP that were stable and where no ectopic beats were present were selected for analysis. A minimum of 6 segments consisting of 3 during the day and 3 during the night periods were identified for analysis for each animal at each age point sampled. Power spectrums for each 80 s period were generated using a fast Fourier transformation (size 256, Hanning window, final frequency resolution of 0.04 Hz), exported as text into Microsoft Excel, then total power (TP; 0–3 Hz), very low frequency (VLF; 0.04–0.2 Hz), low frequency (LF; 0.25–0.75 Hz), high frequency (HF; 1–3 Hz) power values of HRV were calculated. SBP was then derived from the same segments used to estimate HRV, uniformly resampled at 10 Hz and a power spectrum generated (size 256, Hanning window, final frequency resolution of 0.04 Hz) with TP (0–3 Hz), VLF (0.04–0.2 Hz), LF (0.25–0.75 Hz) and HF (1–3 Hz) SBPV power calculated. Similarly, BRS was estimated on the same 80 s segments of AP as HRV and SBPV, using cross-spectral analysis of LF and HF HRV and SBPV power as previously described^24^. A single average was created from all the 80 s segments per age point studied for the final analysis.

#### Biochemical analysis

Renal function was determined by measurement of plasma urea, plasma creatinine, and estimated CCR, as surrogate for glomerular filtration rate. As a measure of filtration barrier injury, urine protein was determined and normalised to urine creatinine level, expressed as urine protein to creatinine ratio (UPC). For urine collection, animals were acclimatised to metabolic cages, and then held for up to 5 h (from 9 am to 2 pm) to collect spot urine samples (Study 1) or for 24 h (from 9 to 9 am (Study 2a and 2b), during which time 24 h urine output and 24 h water intake were measured. Animals were provided free access to food and water in metabolic cages. Urine samples were spun and analysed immediately or frozen until analysis.

At the termination of the study period, animals were euthanised as described above and blood (3–4 ml) was collected via cardiac puncture into ethylenediaminetetraacetic acid (EDTA) tubes (Becton, Dickinson and Company, NJ, USA). The samples were spun and the plasma was extracted, snap frozen in dry ice then stored at − 80 °C until further analysis. Plasma and urine samples were analysed using an IDEXX VetLab analyser (IDEXX Laboratories Pty Ltd., NSW, Australia). CCR was estimated using the following equation:

$${\text{Creatinine}}\,{\text{clearance}} \, {\text{rate }}\left( {{\text{ml/min}} } \right) = \frac{{24\;{\text{h}} \, {\text{urine}} \, {\text{output}} \, \left( {{\text{ml}}} \right) \times {\text{urine}} \, {\text{creatinine}} \, {\text{level}} \, \left( {\upmu {\text{mol/L}}} \right)}}{{{\text{plasma}} \, {\text{creatinine}} \, {\text{level}} \left( {\upmu {\text{mol/L}}} \right) \, \times 24 \times 60}}.$$

#### Urine sodium analysis

Urine sodium from animals in Study 2b (n = 13 Lewis and n = 12 LPK) were analysed using the 4100 MP-AES system (Agilent Technologies, USA), a microwave plasma atomic emission spectrometry-based technique, with urine samples diluted to 1:1000 (Lewis) and 1:500 (LPK) in distilled water (dH_2_O). Data acquisition parameters of sample uptake time (20 s), stabilisation time (20 s) and read time (20 s) were used. All parameters, including sample dilution, were optimised in a series of preliminary experiments. Analysis consisted of washing the system with distilled water for 60 s and then running a series of procedural blanks, calibration sodium samples diluted to a range of 0.625 to 10 mg/l using sodium standards (Sigma-Aldrich) and the diluted urine samples sequentially. A repeat analysis of calibration samples was then performed to ensure the accuracy and precision of the procedure. For each sample, five readings were obtained and averaged to create a final concentration reading in mmol/l. Urine sodium concentrations were then normalised using 24 h urine volume and expressed as mmol/24 h.

#### Plasma and kidney renin content determination, and kidney tissue real-time quantitative reverse transcription polymerase chain reaction (RT–qPCR) analysis

The renin content from plasma and kidney samples was determined by measuring the amount of Ang I generated per hour from added substrate using radioimmunoassay undertaken by ProSearch International Australia (Malvern, Vic., Australia). For plasma, blood samples collected via cardiac puncture as detailed above were spun and 1–1.5 ml plasma extracted and stored at − 80 °C until analysis. A total of 2.5µL of plasma was diluted to 25 µl with dH_2_O for subsequent incubation. Frozen kidney tissues were thawed and a representative sample of 0.2 to 0.5 g tissue obtained. This was homogenised in 5 mM EDTA Na_2_ (1/10 wt gram/vol mL) using a BIAOMA FJ-50 homogeniser/blender for 30 to 60 s at ½ speed at 0 °C. Homogenates were immediately centrifuged at 1000×*g* for 10 min, supernatant collected and left at 0 °C for 1 h and then frozen at -20 °C. The supernatant was diluted with dH_2_O or 0.9% saline 1/1000 and 25 µl (equivalent of 0.025 µl) used for subsequent incubation.

The prepared plasma and kidney samples were then incubated at 37 °C with 25 µl of nephrectomised sheep plasma and 25 µl of an angiotensinase inhibitor cocktail containing 30 mM Na_2_EDTA, 3 mM 2,3-dimercaptopropanol and 6 mM 8 hydroxy quinoline buffered with 150 mM sodium phosphate buffer pH 6.2. In preliminary experiments, varying volumes of sample were incubated for varying times to ensure that reaction kinetics were linear and not substrate limited. Subsequent incubations at 37 °C were for 1 h and were terminated by the addition of 925 µl dH_2_O and placing the tubes into boiling water for 4 min. The Ang I generated was measured by radioimmunoassay^53^ and converted to a rate of production known to be linear (zero order kinetics) and expressed as ng Ang I/ml/h (plasma) or ng Ang I/mg/h (tissue) at 37 °C. Intra assay variability (mean, SD and CV) was 93 ± 10.2 ng/ml/h, 11%. Inter assay variability was − 91 ± 11.8, 13%. The IC 50 is at 0.7 ng/ml. Cross reactivity to angiotensin II and angiotensin (1–7) is less than 0.1%.

For intra-renal gene expression analysis, total RNA was extracted from left kidney tissue (30 mg) using the SV total RNA Isolation System (Promega, Wisconsin, USA) and eluted in up to 50 µl of water and stored at − 80 °C. The RNA concentration of each sample was then determined with a Nanodrop 2000 spectrophotometer (Thermo Fisher Scientific, VIC, Australia). The RNA (1 µg) was then reverse-transcribed with the AffinityScript QPCR cDNA synthesis kid (Agilent Technologies, Santa Clara, CA, USA) according to the manufacturer’s instructions. Quantitative-PCR was performed in 20 μl reaction volumes containing 1 μl of cDNA mix, Applied Biosystems PowerUp SYBR Green Master Mix (Thermo Fisher Scientific, VIC, Australia) and gene primers (See Supplementary Table S2) using the ViiA 7 Real-time PCR system (Thermo Fisher Scientific, VIC, Australia). A preliminary primer concentration assay was carried out to determine the optimum primer concentration [providing the lowest mean threshold cycle (Ct) value] for subsequent experiments. An additional preliminary analysis was then undertaken to determine the optimal pair of housekeeping genes, using NormFinder software (https://www.moma.dk/normfinder-software^54^), by identifying the pair with the most stable expression across the two animal strains in kidney tissue. Conditions for qPCR were as follows: 20 s at 95 °C, 20 s at 60 °C, and 15 s at 95 °C, 1 min at 60 °C, and 15 s at 95 °C for 40 cycles, then held at 4 °C. No reverse-transcriptase cDNA mix (− RT) and no template controls were also run on each plate to ensure experiment fidelity. Following qPCR cycling, the products were validated by melt curve analysis.

For relative gene expression level determination, the average Ct value of three replicates for each animal was normalised to the expression of two housekeeping genes β-actin and cytochrome C1. Values were then normalised to housekeeping genes (ΔCt values). ΔCt values were used for subsequent statistical analysis. For determination of fold difference in gene expression between treatment groups within each strain or between two strains, the 2^−(ΔΔCt ± Standard Error (SE) of the ΔCt)^ method was used^55^. To analyse a strain effect, data was presented as a relative expression of the gene of interest in Lewis animals being the reference group. To analyse a treatment effect within each strain, data was presented as a relative expression of the gene of interest with sham animals being the reference group.

### Immunohistochemistry

#### CGRP/TH

After euthanasia, the kidneys and hearts of all animals used in this study were collected and weighed. Kidneys were then post-fixed in 10% neutral buffered formalin or 4% paraformaldehyde for 5 h at 4 °C, washed three times with phosphate buffered saline (PBS), and stored in 30% sucrose for cryoprotection. Then 14 µm coronal kidney sections were cut using a Leica CM1950 cryostat and mounted on superfrost plus slides (Lomb Scientific Pty, Ltd, NSW, Australia). The sections were incubated in blocking solution containing either 20% normal rat serum (v/v, Sigma, USA), 20% donkey serum (v/v, Sigma, USA) (for CGRP immunohistochemistry) or 10% donkey serum (for TH immunohistochemistry), as well as 0.3% Triton-X 100 and 0.05% thimerosal (Sigma, USA) in Tris phosphate buffered saline (TPBSm, 10 mmol/l Tris buffer, 0.9%NaCl, 10 mmol/l phosphate buffer -NaH_2_PO_4_/Na_2_HPO_4_, pH 7.4) at room temperature for 2 h, followed by 48 h incubation in the same blocking solution containing the primary antibody against CGRP or TH (as detailed in Table 5). The specificity of both antibodies has been validated previously^56,57^. A Cy3-conjugated species-specific secondary antibody (as detailed in Table 5) was used to visualise the signal. Kidney sections incubated in sample blocking solution omitting the primary antibody were used as negative controls. Sections were viewed using a ZENPRO epifluorescence microscope (Zeiss, Gottingon, Germany).

**Table 5:** Primary and secondary antibodies used for kidney immunohistochemistry.

Primary antibodies	Dilution and source	Secondary antibodies	Antibody target/marker
Goat anti rat calcitonin gene-related peptide (CGRP) antibody	1:500, Bio-Rad/AbD Serotec Cat# 1720–9007, RRID: AB_2290729	Jackson ImmunoResearch Labs Cat# 715–165-151, RRID: AB_2315777	CGRP
Monoclonal mouse anti-Tyrosine Hydroxylase (TH) antibody	1:200, Avanti Antibodies Cat# AV1, RRID: AB_2531895	Jackson ImmunoResearch Labs Cat# 705–165-147, RRID: AB_2307351	TH
Polyclonal goat anti-renin antibody, IgG isotype	1:500, Santa Cruz, Cat# sczsc27318, RRID: AB_2301005	Jackson ImmunoResearch Labs Cat# 705–165-147, RRID: AB_2307351 or Jackson ImmunoResearch Labs Cat# 705–486-147, RRID: AB_2616594	Renin
Polyclonal rabbit anti-aquaporin 1 (AQP1)	1:100, Alomone lab, Cat# AQP-001. RRID: AB_2039726	Jackson ImmunoResearch Labs Cat# 711–166-152, RRID: AB_2313568	Proximal convoluted tubule and thin descending limb of Henle’s loop
Polyclonal rabbit anti-AQP2	1:2000, Alomone lab, Cat# AQP-002. RRID: AB_2039728	Jackson ImmunoResearch Labs Cat# 711–166-152, RRID: AB_2313568	Cortical and medullary collecting ducts
Fluorescein-labelled peanut Agglutinin (PNA)	1:2000, Vector Laboratories Cat# FL-1071, RRID: AB_2315097	NA	Distal convoluted tubule

Because renal sensory nerves are almost exclusively located in the renal pelvis^20,30^, for quantification of CGRP staining, positive CGRP labelling in the renal pelvic wall was determined using ImageJ software from three sections chosen to contain a significant region of the pelvic wall and averaged for each animal, expressed as percentage of positive staining within the pelvic wall as previously described^28^. Images were thresholded to differentiate positive labeling from background staining, and the percentage of area with positive labeling was then determined after manually outlining the area of the pelvic wall. Due to cystic lesions in LPK animals, methods of TH analysis which determine innervation density as relative area of staining (µm^2^/mm^2^) for kidney regions^49^ could not be applied. Instead, the level of TH staining was quantified in the immediate vicinity of blood vessels in the cortico-medullary junction (a region well established to be high in sympathetic innervation^30^), using a minimum of 4 arteries from each animal based on previous studies^29,13,28^. Innervation was measured no further than 30 µm from the vessels, incorporating both adventitial fibers and associated perivascular nerve bundles but excluding the vascular wall, and expressed as percentage of positive staining within perivascular area. Blind analysis was not possible as the main researcher (SL) performing the analysis was also aware of the group assignment. A minimum of 4 animals per treatment group per strain at each time point (one week and 4 weeks post RDN) were analysed.

#### Renin/tubule markers

Following cryosection as described above, kidney sections were incubated for 2 h at room temperature in blocking solution containing 10% donkey serum in TPBSm as detailed above. Sections were then incubated with primary antibody for renin, in combination with one of the tubule regional markers (Table 5) for 48 h at 4 °C, followed by incubation with species-specific fluorescence-conjugated secondary antibodies for 4 h at room temperature, with the exception of fluorescein-labelled peanut agglutinin (PNA), which did not require a secondary antibody for signal visualisation. The primary and secondary antibodies were diluted with TPBSm containing 10% donkey serum and 0.3% Triton-X 100. Again, negative controls were kidney sections incubated in the blocking solution omitting the primary antibody. Preliminary experiments were carried out to determine the optimal concentration of each primary antibody.

Sections were viewed using a ZENPRO epifluorescence microscope (Zeiss). Representative areas labelled with renin and/or tubule markers was imaged using either 10 × or 20 × objective depending on the size of the region of interest. A total of three animals from each strain were imaged.

### Statistical analysis

Data analysis was performed using GraphPad Prism (v7.02, GraphPad Software, La Jolla, CA, USA) and IBM Statistical Package for the Social Sciences (v25, SPSS; Chicago, IL, USA). Results are expressed as mean ± SEM when appropriate. Preliminary analysis of the data included testing for normality using an Anderson–Darling test.

Immunohistochemical labeling between the three RDN groups within each strain was analysed using one-way ANOVA with Bonferroni’s post hoc test when needed. When comparing morphometric and biochemical parameters for both 7-week and 10-week old cohorts, two-way ANOVA was used with strain and treatment as the two variables. When comparing cardiovascular and autonomic function parameters, a two-way ANOVA was used with treatment and age as the independent variables within each strain. If either a treatment or age effect was noted, the data were further analysed using a Bonferroni’s post hoc test to determine group differences. If data was not normally distributed, it was analysed using a nonparametric Kruskal–Wallis test.

For comparison of kidney and plasma renin content and gene expression analysis, a univariate general linear model analysis of variance was used to identify strain or treatment effects followed by Tukey post hoc analysis as indicated. If the data displayed unequal variance (Levene’s Test), Games-Howell post hoc analysis was used.

Significance was defined as a P value < 0.05 for all analysis.

## Supplementary Information

Supplementary Information.
