# Supplementary material for: Renal denervation does not affect hypertension or the renin-angiotensin system in a rodent model of juvenile-onset polycystic kidney disease: clinical implications
Source: Sci Rep. 2021 Jul 12;11:14286. doi: 10.1038/s41598-021-93575-0 (PMC8275789; doi:10.1038/s41598-021-93575-0)
Supplement: Supplementary file 1 — Supplementary Information. [file 41598_2021_93575_MOESM1_ESM.pdf]

Renal denervation does not affect hypertension or the renin-angiotensin system in a rodent model of juvenile-onset polycystic kidney disease: clinical implications.

Sheran Li[1], Cara M Hildreth[1], Ahmed A Rahman[1], Sean A Barton[1], Benjamin F Wyse[1], Chai K. Lim[1], Paul M Pilowsky[2], Jacqueline K Phillips[1] ✉

[1]Department of Biomedical Sciences, Faculty of Medicine, Human and Health Sciences, Macquarie University, Sydney, Australia.
[2]Discipline of Physiology, School of Medical Sciences, University of Sydney, Sydney, Australia.

✉Professor Jacqueline K Phillips
Department of Biomedical Sciences, Faculty of Medicine, Health and Human Sciences, Macquarie University, Sydney NSW 2109, Australia
Fax: 61 2 98502701
Phone: 61 2 98502753
Email: jacqueline.phillips@mq.edu.au

Supplementary Figure S1: Flow chart illustrating experimental design

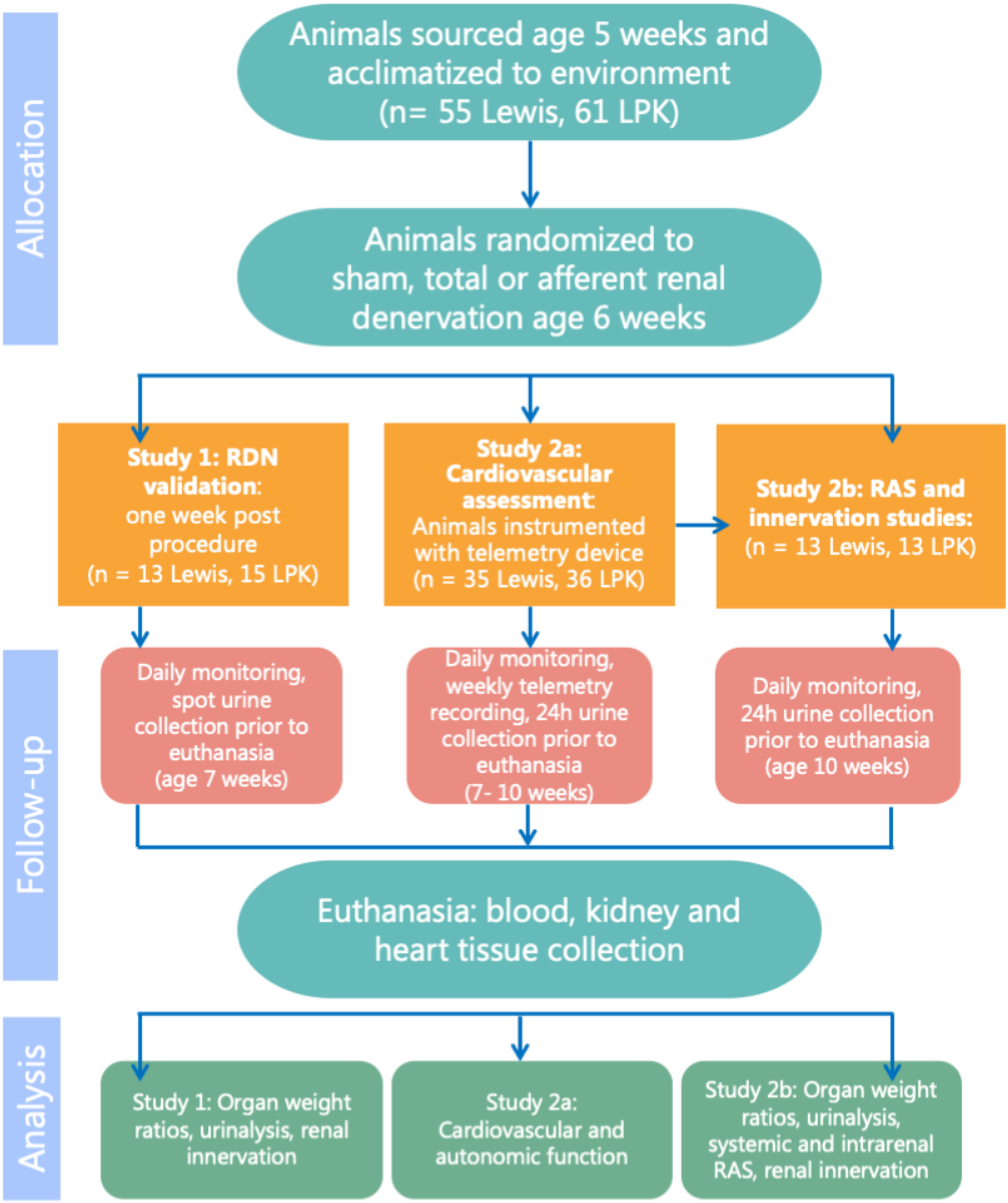

Animals in each study cohort were allocated to each treatment group as indicated at age 6 weeks, then went through follow-up protocols prior to euthanasia, tissue collection and data analysis. Lewis polycystic kidney (LPK).

Supplementary Table S1: Breakdown of animal numbers in each study cohort

Cohort	Lewis male	Lewis female	LPK male	LPK female	Total
**Study 1: 7 wks**					
Sham RDN	4	1	3	3	11
Total RDN	3	1	2	3	9
Afferent RDN	4	0	3	1	8
Total (M/F)	Lewis: 13 (11/2)		LPK: 15 (8/7)		28
**Study 2a: BP data**					
Sham RDN	$6^{(1)}$	$5^{(1)}$	$7^{(1)}$	6	$24^{(3)}$
Total RDN	$8^{(1)}$	$6^{(1)}$	8	6	$28^{(2)}$
Afferent RDN	$5^{(1)}$	$5^{(1)}$	$5^{(2)}$	4	$19^{(4)}$
Total (M/F)	Lewis: 35 (19/16)		LPK: 36 (20/16)		$71^{(9)}$
**Study 2b: Tissue collection**					
Sham RDN	2	3	2	2	9
Total RDN	2	2	2	2	8
Afferent RDN	2	2	3	2	9
Total (M/F)	Lewis: 13 (6/7)		LPK: 13 (7/6)		26

Animals in each study cohort. For Study 2 re BP data: number in superscript indicates number of animals that were also used for tissue collection in Study 2b. Therefore those 9 animals are also counted in Study 2b and overall total number of animals used was 28 + 71 + (26 - 9) = 116. RDN: renal denervation, BP: blood pressure.

Supplementary Table S2: List of primer pairs for RT–qPCR.

		Primer Sequence (5'->3')			
Genes	NCBI Reference	Forward	Reverse	Primer concentration	Size (bp)
Renin	NM_012642.4[1]	CACTCTTGTTGCTCTGGACCT	GGGGTACCAATGCCGATCTC	600nM	250
AGT	NM_134432.2 [2]	CACGGACAGCACCCTATTTT	GCTGTTGTCCACCCAGAACT	600nM	103
ACE1	NM_012544.1 [3]	CACCGGCAAGGTCTGCT	CTTGGCATAGTTTCGTGAGGAA	300nM	97
ACE2	NM_001012006.1 [4]	CCCAGAGAACAGTGGACCAAAA	GCTCCACCACACCAACGAT	300nM	64
Agtr1a	NM_030985.4 [5]	CACAGTGTGCGCGTTTCAT	GTAAGGCCCAGCCCTATGG	300nM	63
β-ACT	NM_031144.3 [6]	GGTCCACACCCGCCACCAG	CGATGGAGGGGAAGACGGC	300nM	128
CYC-1	NM_001277194.1	GCATGGCTCCTCCCATCTAC	CCCATGCGTTTTCGATGGTC	300nM	139

AGT, angiotensinogen; ACE, angiotensin-converting enzyme; Agtr1α, angiotensin type 1α receptor; β-ACT (β-actin); CYC1, cytochrome c1

Supplementary Figure S2

The effect of sex on systolic (SBP; A,B) diastolic blood pressure (DBP; C, D) and heart rate (HR; E, F) in Lewis (A, C and E) and LPK (B, D and F) rats between 7-10 weeks of age.

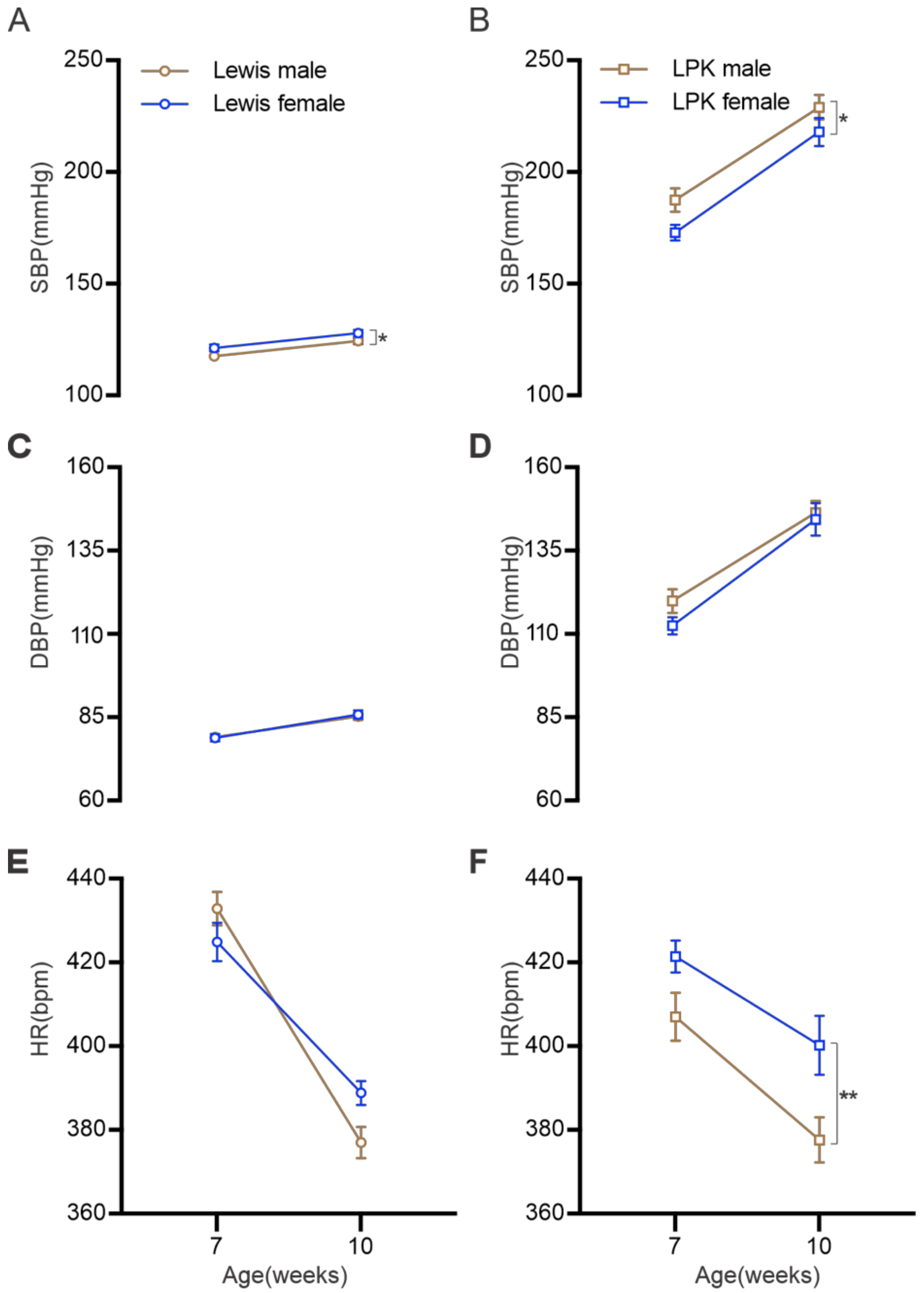

N values at each week for Lewis male = (18, 18); Lewis female = (15, 16), LPK male = (20, 16) , LPK female = (16, 15) respectively. Data is expressed as mean ± SEM and analysed with two-way ANOVA for sex and age effect.

Female Lewis had a significantly higher level of SBP but comparable level of DBP and HR relative to male Lewis. Male LPK had a significantly higher level of SBP and lower level of HR compared to female LPK. *indicates P<0.05 and ** indicates P < 0.01 difference between male and females, analysed using two-way ANOVA and Bonferroni's post hoc analysis.

Supplementary Figure S3

The effect of day and night period on systolic (SBP; A, B), diastolic blood pressure (DBP;C, D) and heart rate (HR; E, F) in all Lewis (A, C and E) and LPK (B, D and F) rats between 7-10 weeks of age.

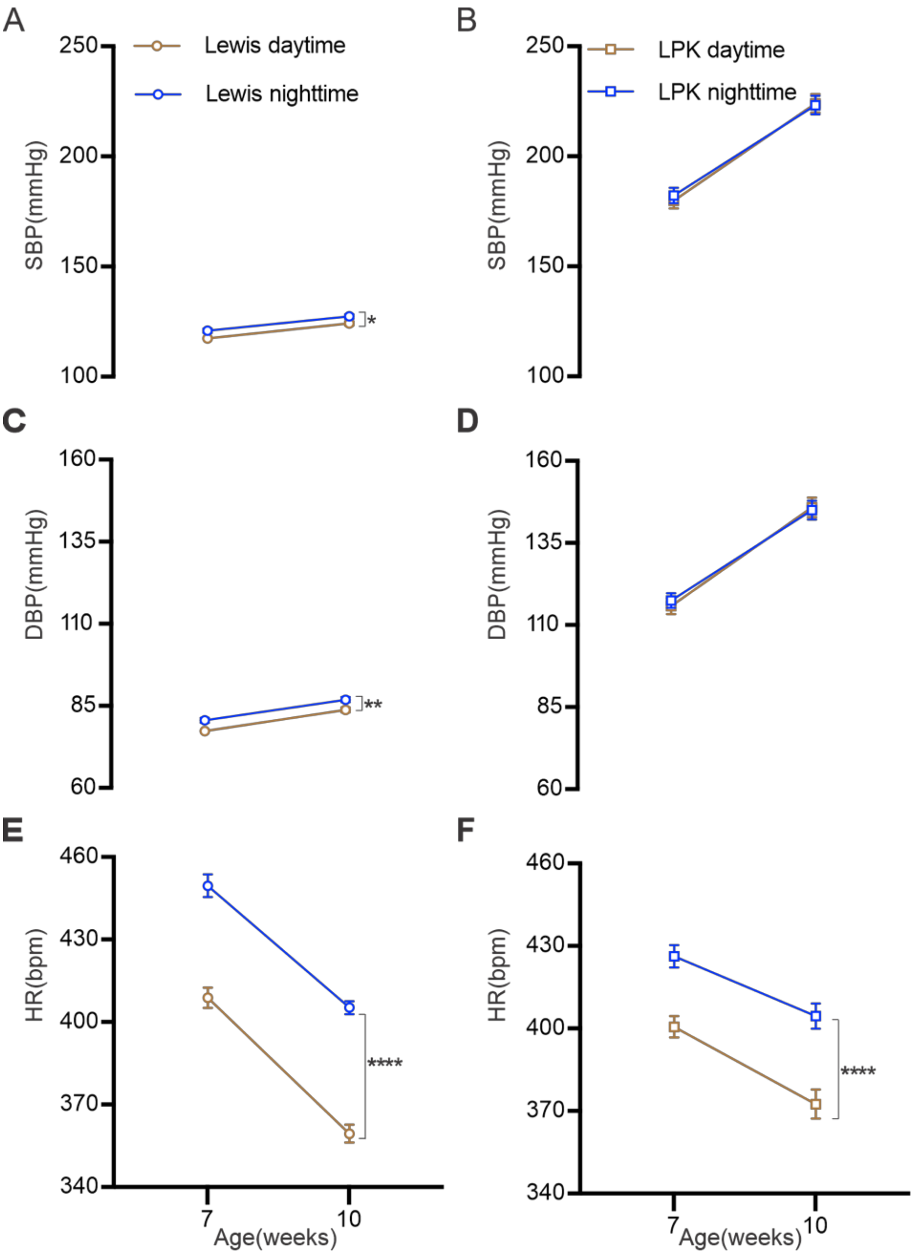

N values at each week for Lewis = (33, 34); LPK = (36, 31) for both day and night values, respectively. Data is expressed as mean ± SEM and analysed with two-way ANOVA for day period and age effect.

Lewis rats had a significantly higher level of SBP, DBP and HR during night-time compared with daytime. LPK rats had a significantly higher level of HR during night-time vs. daytime, but the level of SBP and DBP were comparable during both periods, suggesting a non-dipping status. *indicates P<0.05, ** indicates P < 0.01 ****indicates P<0.0001 difference between daytime and night-time values, analysed using two-way ANOVA and Bonferroni's post hoc analysis.

Supplementary Figure S4

The effect of renal denervation (RDN) on the differences between night-time and daytime SBP (ΔSBP, A and B), DBP (ΔDBP, C and D) and HR (ΔHR, E and F) in Lewis (A, C and E) and LPK (B, D and F) rats between 7-10 week of age after sham, total and afferent RDN procedures.

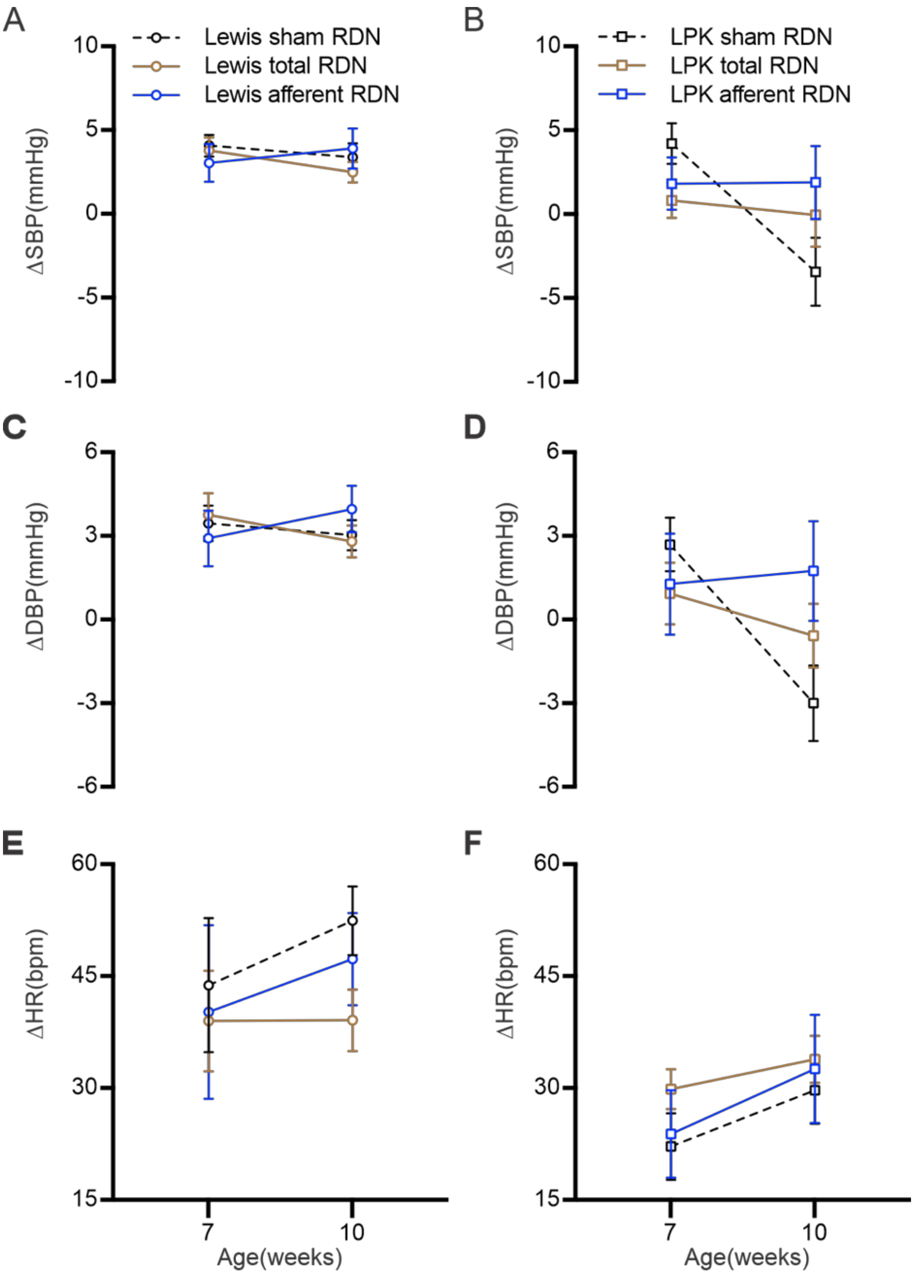

Data is expressed as mean ± SEM, analysed using Two-way ANOVA. N values at each week for Lewis sham = (10, 11); Lewis total = (14, 13); Lewis afferent = (9, 10) respectively and for LPK sham = (13, 11), LPK total = (14, 12) and LPK afferent = (9, 8), respectively.

No overall treatment effect was present for any of these parameters in either strain.

References

1 Williamson, C. R., Khurana, S., Nguyen, P., Byrne, C. J. & Tai, T. C. Comparative analysis of renin-angiotensin system (RAS)-related gene expression between hypertensive and normotensive rats. *Med Sci Monit Basic Res* **23**, 20-24, (2017).

2 Chung, S. *et al.* Tempol or candesartan prevents high-fat diet-induced hypertension and renal damage in spontaneously hypertensive rats. *Nephrol. Dial. Transplant.* **25**, 389-399, (2010).

3 Paizis, G. *et al.* Up-regulation of components of the renin-angiotensin system in the bile duct-ligated rat liver. *Gastroenterology* **123**, 1667-1676, (2002).

4 Anton, L. *et al.* Activation of local chorionic villi angiotensin II levels but not angiotensin (1-7) in preeclampsia. *Hypertension* **51**, 1066-1072, (2008).

5 Sanguesa, G. *et al.* Type of supplemented simple sugar, not merely calorie intake, determines adverse effects on metabolism and aortic function in female rats. *Am. J. Physiol. Heart Circ. Physiol.* **312**, H289-H304, (2017).

6 Colegio, O. R. *et al.* Functional polarization of tumour-associated macrophages by tumour-derived lactic acid. *Nature* **513**, 559, (2014).
